# Remote heat dissipation in atom-sized contacts

**DOI:** 10.1038/s41598-018-26203-z

**Published:** 2018-05-18

**Authors:** Makusu Tsutsui, Takanori Morikawa, Kazumichi Yokota, Masateru Taniguchi

**Affiliations:** The Institute of Scientific and Industrial Research, Osaka University, 8-1 Mihogaoka, Ibaraki, Osaka 567-0047 Japan

## Abstract

Understanding and control of heat dissipation is an important challenge in nanoelectronics wherein field-accelerated hot carriers in current-carrying ballistic systems release a large part of the kinetic energy into external bulk phonon baths. Here we report on a physical mechanism of this remote heat dissipation and its role on the stability of atomic contacts. We used a nano-fabricated thermocouple to directly characterize the self-heating in a mechanically-configurable Au junction. We found more pronounced heat dissipation at the current downstream that signifies the electron-hole asymmetry in Au nanocontacts. Meanwhile, the simultaneously measured single-atom chain lifetime revealed a minor influence of the heat dissipation on the contact stability by virtue of microleads serving as an effective heat spreader to moderate the temperature rise to several Kelvins from the ambient under microwatt input power. The present finding can be used for practical design of atomic and molecular electronic devices for heat dissipation managements.

## Introduction

Energy dissipation in a current-carrying nanosystem is a fundamental yet intriguing issue for the goal of nanoelectronics^1–4^; whereas electrical heating in bulk structure is described unambiguously by Joule-Lenz law^5^, where field-accelerated electrons release heat homogeneously in the material via scattering with phonons, self-heating in nanoscale conductors having characteristic size smaller than the electron mean free-path involves ballistic transmission of charge carriers thereby entailing an unusual interplay between electrical heating and concomitant lattice cooling by heat conduction to the leads. More specifically, despite in a ballistic limit, electrons still have a finite probability for being scattered by phonons in the nanostructure and yield non-negligible amount of heat there^6,7^. Due to the huge current density, this local heating was found significant enough to raise the effective temperature of the quasiballistic contact and severely degrades the stability in cases when phonon coupling to the bulk is inefficient^8–10^.

**Figure 1:**
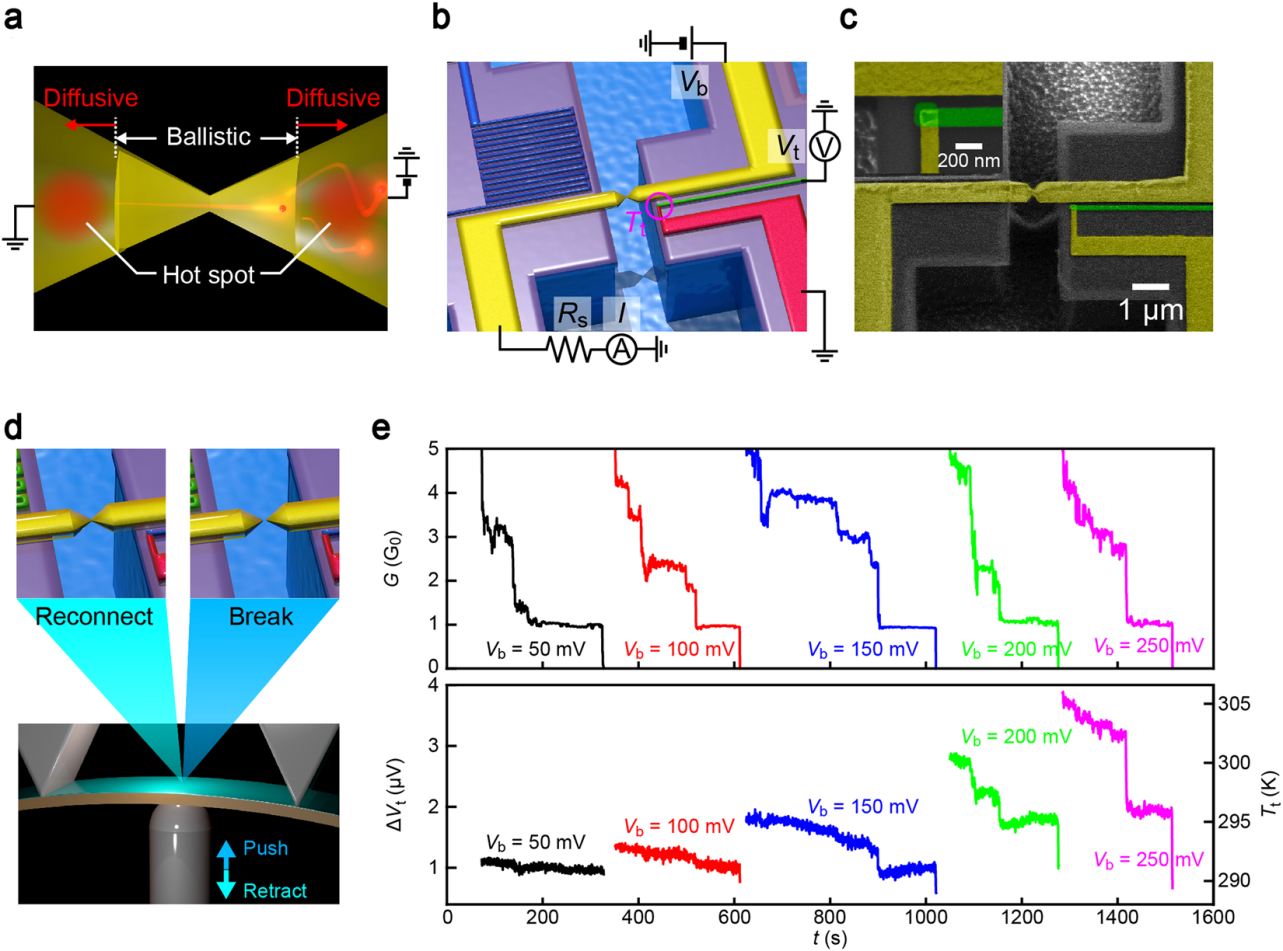
Detections of remote heat dissipation. (**a**) Schematic illustration of heat dissipation in a ballistic Au nanocontact. (**b**) Measurement circuit used to investigate remote heat dissipation effects. The thermovoltage at the embedded Au/Pt thermometer was recorded while mechanically breaking the Au contact under the applied voltage *V*_b_. (**c**) Scanning electron micrograph of a thermometer-embedded mechanically-controllable break junction. Inset shows a magnified view of the point contact of the thermocouple comprised of Au and Pt nanowires. (**d**) Break junction mechanism. The substrate was bended to break/reconnect the Au junction repeatedly under a conductance feedback control. (**e**) Conductance (upper) and thermovoltage traces (lower) obtained simultaneously during contact elongation under various *V*_b_ conditions ranging from 50 mV to 250 mV.

Meanwhile, in contrast to the fact that only a marginal portion of the electrical power is consumed in the on-site local heating, the majority is dissipated at tens of nanometers away from the ballistic system by inelastic scattering of hot electrons whereby creating hot spots (Fig. 1a)^11–13^. This remote heat dissipation has been recently investigated for atomic and molecular junctions by measuring local temperature at the electrode bank^14,15^. Intriguingly, the energy dissipation in the essentially symmetric nanostructure was observed to be asymmetric with respect to the applied voltage polarities depending on the transmission characteristics at the Fermi level^14–16^. However, whether the heat generated at the electrodes contributes to the current-induced instability of nano-junctions has yet to be explored, which is of importance for developing practically viable nano-dimensional building blocks for future nanoelectronic devices. In this study, therefore, we evaluated the influence of the remote energy dissipation on stability of ballistic atom-sized junctions at room temperature in vacuum by simultaneously measuring the contact lifetime and the electrode temperature.

## Result and Discussion

### Simultaneous measurements of junction conductance and temperature

A mechanically-stable experimental system is a prerequisite for the evaluation of contact stability. Here we employed a microfabricated mechanically-controllable break junctions (MCBJs) designed to have the displacement ratio *r* = 6*ut*/*L*^2^ = 3 × 10^−4^ for the natural lifetime measurements of Au atomic contacts at room temperature, where *u*, *t*, and *L* are the free-standing length of the metal nanobridge, the thickness of an MCBJ substrate, and the distance between the two counter-supports, respectively. We lithographed a thermocouple on the MCBJ to create Au nanocontacts by substrate bending and at the same time detect the electrical heat at one side of the contact bank under current flow (Fig. 1b,c). In experiments, Au junctions were gradually stretched and broken under substrate deflection control using a piezo-actuator. During the mechanical breaking processes, temporal change in the contact conductance *G* under the applied dc voltage *V*_b_ and the thermovoltage at the thermocouple *V*_t_ were recorded. Here, a special care was taken to stop the elongation when *G* decreased to below 6 *G*_0_ (*G*_0_ = 2e^2^/*h* is the conductance quantum where *e* and *h* are the electron charge and Planck’s constant, respectively) and let the atom-sized contacts break spontaneously via thermal fluctuations for estimating the natural lifetime^17,18^. After that, the contacts were reconnected by reversing the bending motion (Fig. 1d). The course of break/connect processes were implemented repeatedly for 100 cycles.

Figure 1e shows *G* and Δ*V*_t_ versus time traces at *V*_b_ ranging from 0.05 V to 0.25 V. In response to the tensile forces imposed through the substrate bending, the junction conductance was observed to decrease in a step-wise fashion representing the contact mechanics at the narrowest constriction involving elastic elongation and atomic rearrangement processes. Meanwhile, the simultaneously recorded *V*_t_ changed in a similar manner almost completely tracing the discrete drops in *G*. We also observed that the thermovoltage feature became more pronounced with increasing *V*_b_, which is naturally interpreted as suggesting more significant Joule heat generated under higher input power that indirectly elevates the local temperature at the thermocouple.

### Au contact size dependence of heat dissipation

**Figure 2:**
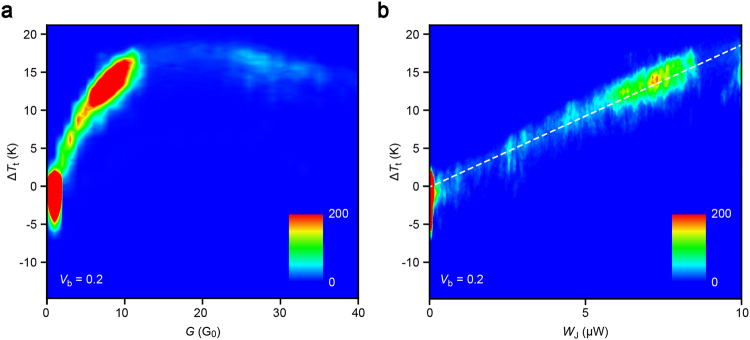
Power-dependent remote heat dissipation. (**a**,**b**) Two dimensional histograms of the change in temperature at the thermocouple Δ*T*_t_ from the ambient plotted as a function of the junction conductance *G* (a) and the input power *W*_J_. Dashed line is a linear fit to the plots.

With the calibrated nanoscale thermometer, with which *V*_t_ can be converted to the local temperature *T*_t_ at the thermocouple point contact (see Supplementary Information Figs S1–S3), we analysed possible contact size effects on the remote heat dissipation from nano- to single-atom scale by examining the junction conductance dependence of the change in thermocouple temperature Δ*T*_t_ from the ambient in order to first explore the heat dissipation mechanism in the current-carrying nanocontacts. It is first noted that in a range of conductance studied (*G* = 1 *G*_0_ to 40 *G*_0_), the constriction diameter *d* is smaller than 2.2 nm according to Sharvin’s formula^19^ predicting *G* = (2*e*^2^/*h*)(*k*_F_*d*/4)^2^ where *k*_F_ = 1.2 × 10^10^ m^−1^ is the Fermi wavenumber for bulk Au^19^. As the inelastic mean free path of Au (~38 nm)^20^ far exceeds *d* here, most of the input power is expected to dissipate at locations far away from the atomic-scale constriction^12^. A two-dimensional histogram of Δ*T*_t_ and *G* (Fig. 2a) displayed a peculiar characteristic wherein the temperature took a local maximum indicating cooling of the Au junction under larger power transmission at *G* > 20 *G*_0_. This is explained by considering the influence of the protection resistance *R*_s_ of 1 kΩ connected in series to the Au leads (Fig. 1b). At the high conductance regime of *G* > 10 *G*_0_, the applied voltage is divided by more than 40% on the serial resistor making the effective voltage *V*_J_ loaded on the Au junctions much lower than *V*_b_ by a factor of *R*_J_/(*R*_J_ + *R*_s_), where *R*_J_ = *G*^−1^. Taking this effect into account, we plotted Δ*T*_t_ as a function of the effective power *W*_J_ input on the Au wire (Fig. 2b), which revealed linear behaviour suggestive of heat dissipation by release of a large portion of the electron kinetic energy into the phonon baths at several tens of nanometer away from the ballistic contact^15^. The fact that the linear dependence extends from nanoscale down to the single-atom level elucidates no notable contact size effect in the remote heat dissipation unless it is in a ballistic condition; in other words, the number of hot charges created per time solely determines the amount of heat generation at the bank under constant bias.

### Dynamic response of thermocouple temperature

Close inspection of the conductance and thermovoltage traces reveal not only the average dependence but also the dynamic response of Δ*T*_t_ on the input power. By directly comparing Δ*T*_t_ and *W*_J_ in individual traces, we see complete synchronization between the two properties (Fig. S4) manifesting much faster relaxation of the system to a steady state^21^ than the temporal resolution of the measurement circuit of 0.3 s.

### Asymmetry in remote heat dissipation

**Figure 3:**
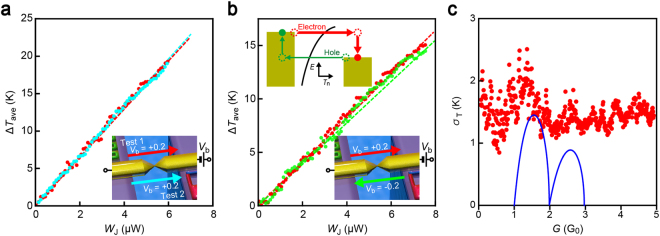
Bias polarity dependence of remote heat dissipation. (**a**,**b**) Δ*T*_ave_ versus *W*_J_ scatter plots (solid circles) and linear fitting (dotted lines) for two tests under the same *V*_b_ condition (**a**) and different bias polarity (**b**) showing comparatively smaller temperature increase at the thermocouple under the negative voltage. Inset illustration explains the mechanism responsible for the asymmetric heat dissipation in the Au nanocontacts. (**c**) Fluctuation *σ*_t_ of Δ*T*_t_ plotted as a function of *G*. Solid curves denote *σ*_S_  ~ *T*_*n*_√1 − *T*_*n*_/(*n* − 1 + *T*_*n*_) where *T*_n_ is the transmission probability of the *n*th channel.

It is anticipated a priori that equal amount of energy is dissipated at both sides of the ballistic conductor considering the symmetric structure; and hence the remote heat dissipation is bias polarity independent^12^. To verify this, we first carried out two independent experiments at *V*_b_ =  + 0.2 V consecutively using a heater-embedded MCBJ (Fig. 3a). Specifically, we acquired 100 traces of *G* and Δ*T*_t_ during junction breaking and calculated the average thermocouple temperature change Δ*T*_ave_ with respect to *W*_J_ binned at 10 nW. The results demonstrated little difference in Δ*T*_ave_ versus *W*_J_ characteristics between the trials. In contrast, when investigating the asymmetry in energy dissipation through performing the local temperature measurements by alternately changing *V*_b_ from +0.2 V to −0.2 V after every each break/connect process (Fig. S5), we found slightly larger increase in the thermocouple temperature at the positive voltage condition (Fig. S6) suggesting more significant heat dissipated at the current downstream of the atom-sized junctions (Fig. 3a,b; the difference in Δ*T*_ave_ between the two figures is attributed to a slight variation in the device structure fabricated by the lithography-based processes).

The asymmetric feature in the thermocouple temperature can be explained by Landauer theory, which predicts a difference in the heat dissipation Δ*Q* at current upstream (*Q*_up_) and downstream (*Q*_down_) of a ballistic system as Δ*Q* = (*Q*_up_ − *Q*_down_) ~ 2*GTSV*_b_, where *T* and *S* are the temperature and the Seebeck coefficient, respectively^15,22^. On the other hand, while Au nanocontacts are reported to possess structure-dependent bi-thermoelectric properties^23–25^, previous studies reported negative thermopower of *S*_Au_ = −4 μV/K as an average over many configurations measured^24^. Accordingly, Δ*Q* < 0 from the sign of *S*_Au_, and hence *Q*_down_ > *Q*_up_, that explains the higher Δ*T*_t_ observed under positive *V*_b_. The underlying mechanism involves a higher probability of electron transmission than the hole counterpart in the case for *S*_Au_ ~ −dln(*T*_n_)/d*E* < 0 (Fig. 3b inset) that leads to larger number of field-accelerated hot charges to release the energy at the anode than the cathode.

More quantitatively, *G* is about 0.2 mS when *W*_J_ = 8 μW. At this condition, the heat asymmetry Δ*Q* is estimated to be 90 nW. On the other hand, as *Q*_up_ is about 4 μW, the Landauer model predicts larger heat dissipated at the current downstream by approximately 4%. As *T*_t_ scales linearly with *W*_J_^15^, this can be considered as a fair agreement with 3% difference in Δ*T*_ave_ (Fig. 3b). Although the above discussions are based on the average properties, recent work by Cui *et al*.^26^ reported more rigorous evidence on the intimate relation between the heating asymmetry and the thermopower characteristics on molecular tunnelling junctions^26^.

The virtual thermopower trait of remote heat dissipation can in fact be found in the thermometer temperature fluctuation. Figure 3c shows the standard deviation *σ*_T_ of *T*_t_ plotted against the junction conductance. Interestingly, the plots demonstrated a peak at around 1.3 *G*_0_ suggesting more pronounced fluctuations in the amount of heat dissipated in case when the transmission probability of a current-carrying channel becomes less transparent. This peculiar feature cannot be explained as a result of Johnson-Nyquist noise that should scale with √*T*_t_. Shot noise in Au atomic chains is known to show similar *G*-dependence with deep minima at conductance quanta^27^, but hardly detected at the sampling rate (3 Hz) in the present study^28^. Rather, presuming the thermopower contribution in the remote heat dissipation, or equivalently *T*_t_, this characteristic feature can be interpreted as reflecting thermoelectric power noise *σ*_S_. Previous studies reported significant effects of quantum interference between transmitted and elastically backscattered electrons in the atom-sized contacts on their charge transmission characteristics^29^. The mechanism involves random change in the interference paths in response to the varying scattering sites near the constriction derived from atomic rearrangements during junction stretching whereby causing fluctuations in the energy dependence of transmission, or equivalently the contact thermopower^23,29^. Considering a Au nanocontact as a ballistic system with *n* channels of transmissivity *T*_n_ for electron conduction and two diffusive regions connected at both sides, the interference-derived thermopower fluctuation is described as *σ*_S_ ~ *T*_*n*_√1 − *T*_*n*_/(*n* − 1 + *T*_*n*_). This predicts suppressed thermopower noise at the conductance quanta(solid curves in Fig. 3c)^23,29^. On the other hand, since the remote heat is linearly related to *S*_Au_, *σ*_S_ is also expected to be directly linked to *σ*_T_. The similarity in the *G*-dependence of *σ*_T_ and *σ*_S_ in Fig. 3c thus corroborates the thermopower contributions in the remote heat dissipation.

**Figure 4:**
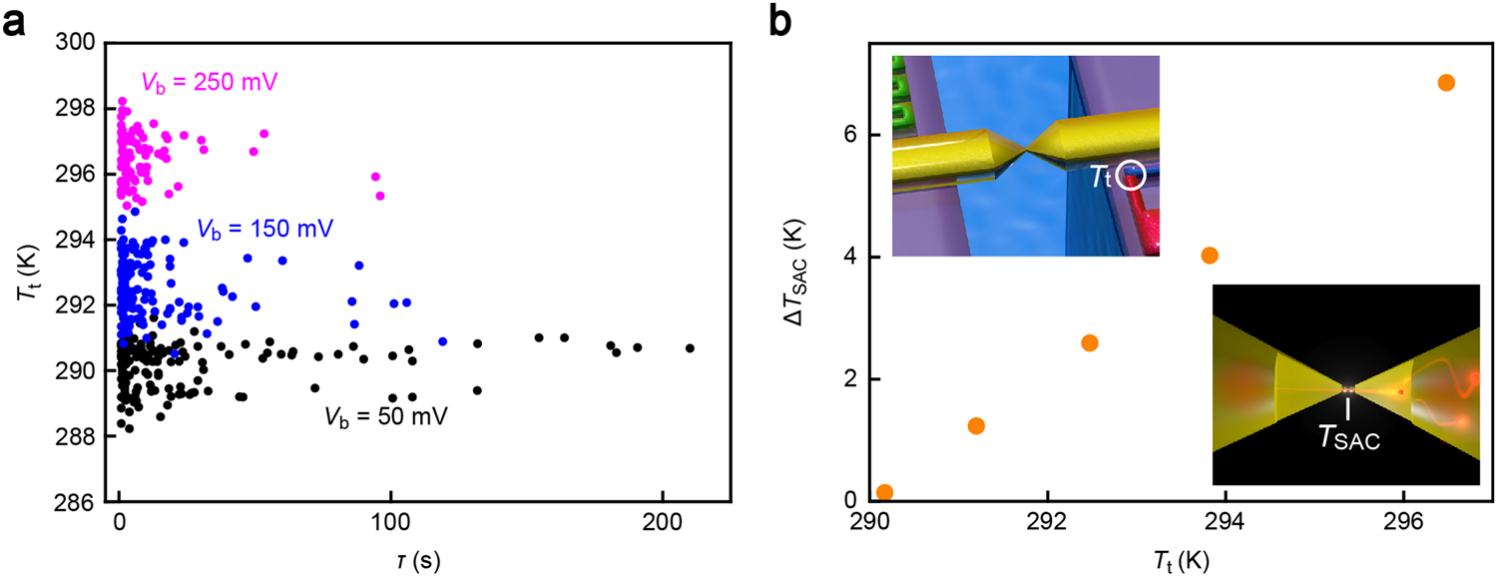
Single-atom contact instability induced by remote heat dissipation. (**a**) Lifetime *τ* of Au single-atom contacts plotted against the thermocouple temperature *T*_t_ obtained under the applied voltage *V*_b_ of 50 mV(black), 150 mV (blue), and 250 mV (purple). (**b**) The single-atom contact effective temperature Δ*T*_SAC_ plotted as a function of *T*_t_.

We now discuss the remote dissipation effects on the stability of atomic contacts. The lifetime *τ* of single-atom chains was deduced by the length of conductance plateaus in a window of 0.8 *G*_0_ to 1.2 *G*_0_. The scatter plots of *τ* with respect to the average *T*_t_ recorded simultaneously with *G* revealed broad distributions in the lifetime (Fig. 4a). This is interpreted as reflecting variations of atomic contact configurations that render a distribution in the barrier energy *E*_B_ against thermal breakdown and resulting exponential scattering described in an Arrhenius form as *τ* = 1/*f*_0_exp(*E*_B_/*k*_B_*T*_SAC_) where *f*_0_, *k*_B_, and *T*_SAC_ are the attempt frequency, the Boltzmann factor, and the effective temperature of single atom contacts, respectively^8,9^. Meanwhile, it is interesting to note that there is an obscure yet distinct tendency where *τ* tends to be longer when *T*_t_ is lower within the data acquired at the same *V*_b_ conditions (Fig. 4a), which suggests a possible role of the energy dissipation on the effective temperature of atomic contacts.

To extract further information from the intriguing result suggesting contact instability by remote heat dissipation, we first assumed the effect in a first order approximation as *T*_SAC_ = *βT*_t_, where *β* is a coefficient describing effects of heat source at the hot spots on the contact temperature (Fig. 4b insets). Then, the lifetime is depicted as a function of *T*_t_ as *τ* = 1/*f*_0_exp(*E*_B_/*k*_B_*βT*_t_). By constructing a least-square fitting to the semi-logarithmic plots of *τ* against the inverse thermocouple temperature 1/*T*_t_, we obtained *β* from the slope amounting 1.1, where *E*_B_ = 0.8 eV was assumed for Au single atom chains (Fig. S7)^30^. The attempt frequency *f*_0_ of 1.6 × 10^12^ Hz was also estimated from the intersect of the fit curve, which is in close accordance with the theoretical values^31^. This implies a small yet finite contribution of the remote heat dissipation to enhance the single-atom contact temperature.

**Figure 5:**
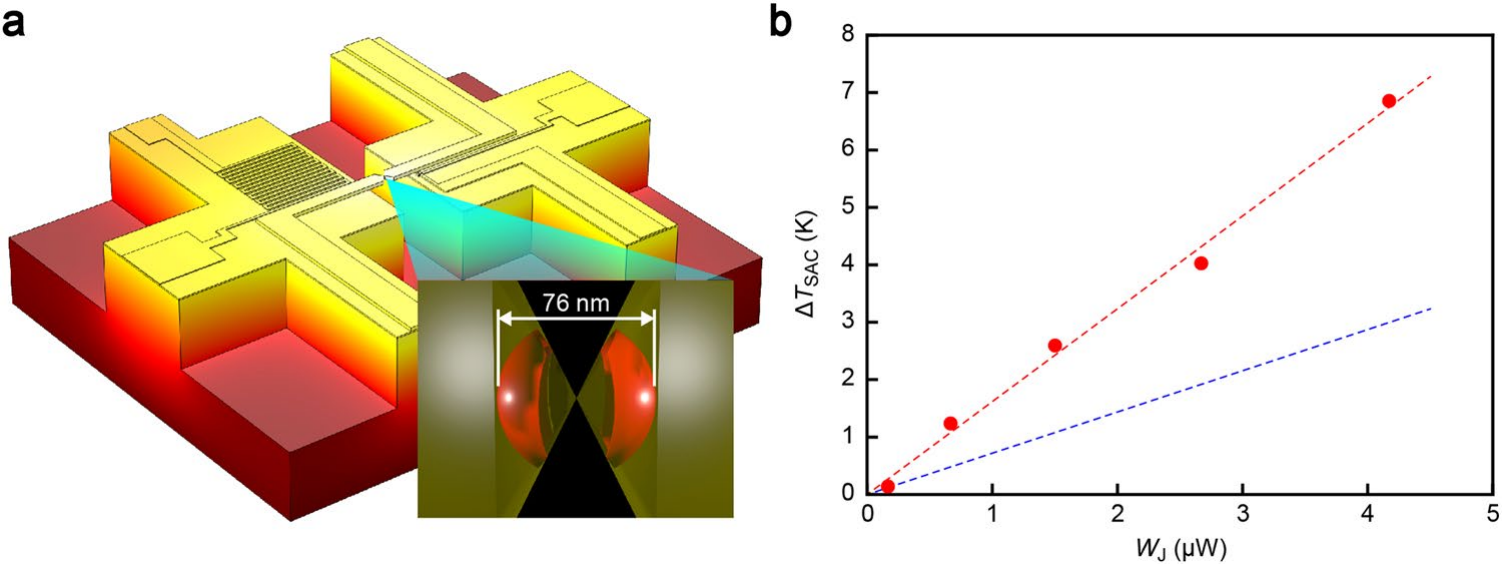
Numerical analysis of remote heat dissipation. (**a**) Temperature profile in the MCBJ structure deduced by a numerical simulation using COMSOL. Two heat sources positioned at both sides of the junction by distance 38 nm emitting heat energy at 5 μW are included in the model (inset) as imaginary hot spots to estimate the remote heating effects. (**b**) Plots of the effective temperature of single-atom contacts *T*_SAC_ as a function of the input power WJ. Red and blue dashed lines are the linear fitting to the plots and the results of numerical simulations, respectively.

The deduced *β* was used to assess the contact temperature from *T*_t_ wherein we found linear rise in *T*_SAC_ with *W*_J_ at a rate 1.6 K/μW. As *T*_t_ ~ *W*_J_ (Fig. 2b), *T*_SAC_ is also linearly dependent on *T*_t_ (Fig. 4b), which is naturally interpreted as denoting direct influence of the remote heat on the single-atom contact stability. In order to verify this, we performed a finite element analysis of thermal dissipation in the Au atomic junction using COMSOL (Fig. 5a). Instead of simulating Joule heat in a current-carrying ballistic contact, we set two heat sources at a distance defined by the inelastic mean free path of 38 nm from the center to mimic the energy dissipation of ballistic electrons (Fig. 5a inset). Here, we presumed slightly larger power dissipation at the thermocouple side of the contact by 3% considering *V*_b_ > 0 V as deduced from the slope *α* of *T*_t_ − *W*_J_ characteristics at positive and negative bias voltage under an assumption of *T*_t_ ~ *Q* (Fig. S4), the difference of which is in fact fair agreement with the Landauer prediction of *α*_*V*b>0_/*α*_*V*b<0_ = (*V*_b_ + *TS*_Au_)/(*V*_b_ − *TS*_Au_) ~ 1.02 with *S*_Au_ = −5.8 μV/K that includes the bulk lead contribution of 1.8 μV/K. The result reproduced the linear increase in *T*_SAC_ with the source power *W*_spot_ at the hot spots (Fig. 5b) but with *T*_SAC_/*W*_spot_ = 0.3 K/μW lower than *T*_SAC_/*W*_J_ obtained in the experiments (Fig. S6). The factor of five discrepancy presumably stems from the assumption of the conceptual hot spots to represent the electron heating effects. The simulation also revealed an important function of the Au microleads as heat sinks to spread the heat to suppress the self-heating-induced instability of atomic contacts (Fig. S8). Although in a framework of Fourier’s theory wherein any quantum effects are being neglected, and hence only qualitative discussion is available at this time, the above result provides device designs for heat dissipation management in nanoelectronic building blocks.

## Conclusions

Heat dissipation in current-carrying ballistic atomic chains and its influence on the stability was studied by using a microthermometer-embedded mechanical break junctions to simultaneously measure the local electrode temperature and the lifetime of Au nanocontacts of variable size from 100 nm down to single-atom scale. We found that the remote heat dissipation is more pronounced at the current downstream of the Au atomic contacts, the asymmetric feature of which is attributed to the larger contributions of field-accelerated hot electrons than the hole counterparts to the heat dissipation due to the broken electron-hole symmetry characterized by the negative quantum thermopower as predicted by Landauer theory. The thermoelectric signature was also present in the standard deviation of the thermometer temperature suggesting an indirect role of quantum interference between the forward and defect-backscattered electrons in the ballistic conductor on the self heating. Most importantly, we revealed a non-negligible influence of the remote electrical heat dissipation that raised the effective temperature of Au single-atom chains by several Kelvins above the ambient under microwatt-level input power. The present finding can be used for practical design of nanoelectronic devices for heat dissipation managements.

## Methods

### Fabrication of thermocouple-embedded MCBJs

Imide precursor was spin-coated on a phosphor bronze substrate and baked for polymerization to form a 5 μm thick polyimide layer. On the polymer, a microelectrode pattern was delineated by photolithography using AZ5206E photoresist. After development, 50 nm thick Au was deposited with 5 nm thick Cr adhesion layer by radio-frequency magnetron sputtering. The residual resist was removed by immersing the chip in DMF overnight followed by sonication whereby formed three pairs of Au microelectrodes each used for the microheater, the thermocouple, and the nanojunction. To fabricate these structures, we first formed 40 nm thick Al_2_O_3_ islands that served as thermal baths by electron beam lithography with ZEP520A, an inductively coupled plasma sputtering, and lift-off in DMF. After that, we prepared a Au/Pt thermocouple on one side of the Al_2_O_3_ layers by forming a Pt nanowire of 100 nm width and 30 nm thickness by electron-beam drawing, the magnetron sputtering, and the lift-off in organic solvent, and subsequently fabricating a Au nanowire of 100 nm width and 50 nm thickness by the same procedures. The two nanowires were jointed at the apexes in a form of a 100 nm sized point contact. A special care was taken to align the positions of the nanowires at several nanometer precision by using a lithographically-defined external markers at the vicinity of the nanostructures. Using the markers, we then formed a Pt coil at the other side of the Al_2_O_3_ islands by the electron-beam lithography-based nanofabrication processes, which served as a microheater for calibrating the thermocouple. In a similar way, we also formed a 100 nm thick Au junction across the two Al_2_O_3_ regions. Finally, the sample surface was exposed to reactive ion etching using oxygen etchant gas to deep-etch the polyimide layer so as to free the Au junction from the substrate and also to supress the thermal leakage in the cross-plane direction.

### Break junction experiments

A thermocouple-embedded MCBJ was mounted on a stage in a three-point bending configuration. The sample chamber was then evacuated. After the vacuum level reached to below 10^−5^ Torr, the MCBJ substrate was bent mechanically by a piezo-driven pushing rod at room temperature under the applied dc voltage *V*_b_ with a picoammeter/source unit (Keithley 6487). In prior to the process, a protection resistor having the resistance *R*_s_ of 10 kΩ was connected in series for preventing electromigration breakdown of fused Au junctions. This led to the Au junction to break at the narrowest constriction where the strain concentrated to induce necking deformation and eventual tensile breakdown. Soon later, the junction was quickly reconnected by completely releasing the bending force through swiftly retracting the rod. Thereafter, the beam bending was feedback controlled with respect to the junction conductance states in order to gradually apply tension to the Au contact to narrow it down to single-atom size. Here, the conductance *G* was recorded by measuring the current *I* through the junction using the picoammeter. After each sampling of the conductance, the thermovoltage *V*_t_ at the thermocouple was acquired using a nanovoltometer (Keithley 2182). The sampling rate as a whole was about 0.3 Hz.

### Thermocouple calibration

The heater temperature was calibrated by sweeping the dc voltage *V*_h_ applied to the Pt coil while recording the output current using another Keithley 6487. Under the same voltage sweep, *V*_t_ was also measured using the nanovoltometer. After that, the sample stage was heated via PID control using a temperature controller that swept the temperature *T*_s_ from 300 K to 500 K. At the same time, the Pt coil resistance *R*_h_ was measured using the picoammeter to clarify the temperature dependence of *R*_h_. From the *T*_s_ − *R*_h_ and *V*_h_ − *R*_s_ characteristics, we deduced the heater temperature *T*_h_ as a function of *V*_h_. Meanwhile, we obtained *T*_h_ − *V*_t_ plots by the *T*_h_ − *V*_h_ and *V*_h_ − *V*_t_ data.

### Numerical Simulation

Heat transport in a thermocouple-embedded MCBJ was numerically simulated using COMSOL. The nanotructure was modelled according to the dimension as observed by SEM. In the calculation, the temperature at the bottom was set to 293 K while setting the microheater temperature *T*_h_ in a range from 300 K to 500 K. A heat transfer in solids module was utilized to 3D-analyze the temperature distribution in the model. Bulk material properties were used for every part including the Au atomic junction.

## Electronic supplementary material

Supplementary Information
