# Supplementary material for: Remote heat dissipation in atom-sized contacts
Source: Sci Rep. 2018 May 18;8:7842. doi: 10.1038/s41598-018-26203-z (PMC5959854; doi:10.1038/s41598-018-26203-z)
Supplement: Supplementary file 1 — Supplementary Information [file 41598_2018_26203_MOESM1_ESM.pdf]

Supplementary Information for

Remote heat dissipation in atom-sized contacts

Makusu Tsutsui, Takanori Morikawa, Kazumichi Yokota & Masateru Taniguchi

The Supplementary Information includes:

1. Calibration of Au/Pt thermocouple (Figs. S1 – S3)

2. Supplementary Figures (Figs. S4 – S8)

3. Supplementary reference

1. Calibration of thermocouple.

It is important to know the actual temperature T_t at the point contact of a thermocouple for assessing the remote heating effects. However, this is not straightforward as the thermovoltage at the Pt/Au point contact reflects the size effects of metal Seebeck coefficient that could even change the sign from the bulk thermopower. For this, we performed calibration measurements using microheater-embedded MCBJs[S1] to convert V_t into T_t (Fig. S1). The procedure involved characterization of the heater temperature rise at the platinum coil T_h under the input voltage V_h through comparing change in the resistance R_h with respect to that recorded under substrate temperature sweep T_s (Fig. S2a-b). As a result, we found linear increase in the heater temperature with V_h^2 suggesting T_h increase by the input power (Fig. S2c). We then Joule-heated the platinum coil heater and recorded the response of V_t at the thermocouple by heat transport via a Au nanobridge having a narrow constriction of 100 nm square cross-section. We acquired $T_h - V_t$ characteristics from these two results (Fig. S2d). Meanwhile, we performed a numerical simulation utilizing a heat transfer in solids module of COMSOL to estimate T_t under different T_h. The model structure built for the thermal analysis had Al_2O_3 islands, a Au junction, a platinum coil, and a Au/Pt thermocouple on a polyimide layer whose dimensions were taken from that deduced from scanning electron micrographs of the MCBJ sample used (Fig. S1). It confirmed negligible thermal leakage through the substrate by virtue of the deep-etched polyimide structure blocking the heat conduction in the cross-plane direction. In contrast, the in-plane thermal transport was found to be quite efficient due to the relatively high thermal conductance of the 100 nm-sized free-standing Au junction conveying the Joule heat from the heater to the thermocouple via the Al_2O_3 bath. From the theoretically estimated T_h - T_t and the experimental $T_h - V_t$, we derived a linear $V_t - T_t$ calibration curve for the bi-metal thermocouple.

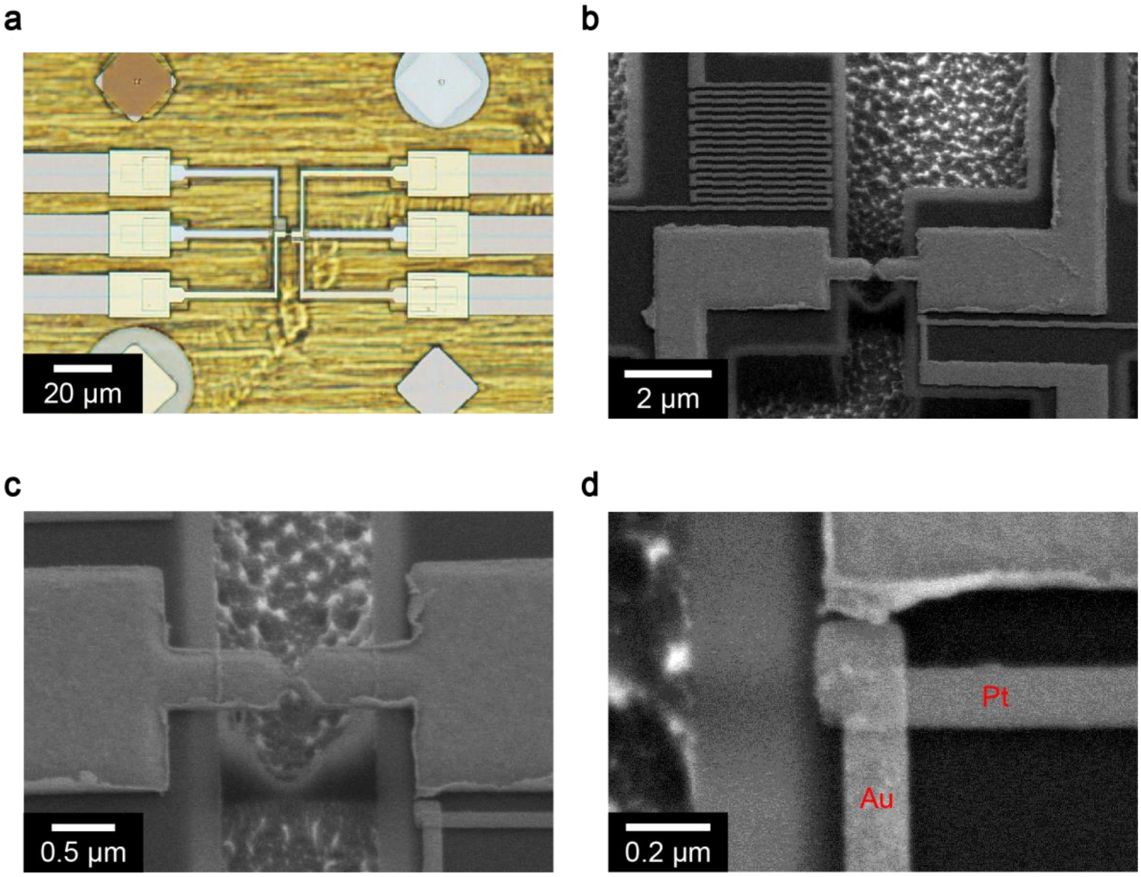

Figure S1. Thermocouple-integrated mechanically-controllable break junctions (MCBJs). **a,** Optical observation of a microheater-embedded MCBJ from top. Four square-shaped patterns were used as external markers for aligning several metal patterns at nanoscale resolution by electron beam lithography. **b,** Scanning electron micrograph of the MCBJ structure. The image was taken with the sample inclined by 45 degrees. Coarse surface is the polyimide sculpted by 2.4 μm via reactive ion etching. Dark contrast shows the 40 nm thick Al_2O_3 islands. On the upper left lies a platinum coil utilized as a heater. At the center, there is a free-standing gold junction bridging the Al_2O_3 layers. The Au and Pt nanowires at the lower right of the image served as a thermometer. **c-d,** Close views of the gold junction (c) and the thermometer (d). The contact area of the nanowires was fabricated to be approximately 100 nm x 100 nm.

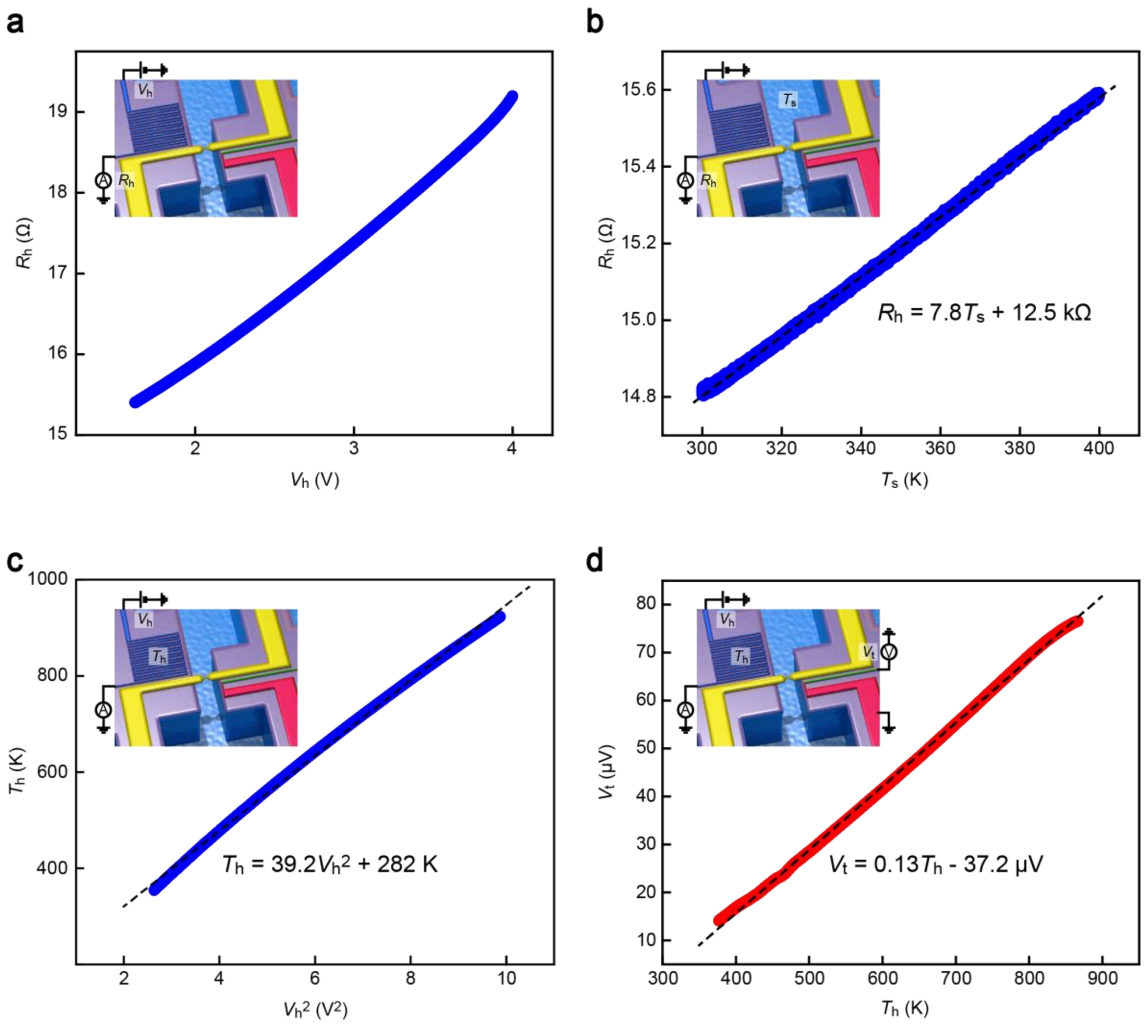

Figure S2. Electrical measurements for thermocouple calibrations. **a-c,** By obtaining the plots of the platinum coil resistance R_h with respect to the applied dc voltage V_h (a) and the substrate temperature T_s (b), we deduced the rate of change in the heater temperature T_h by V_h (c). **d,** Then, the thermovoltage at the thermocouple V_t was measured under a V_h sweep. From the V_h – T_h characteristics, we acquired a T_h – V_t calibration curve.

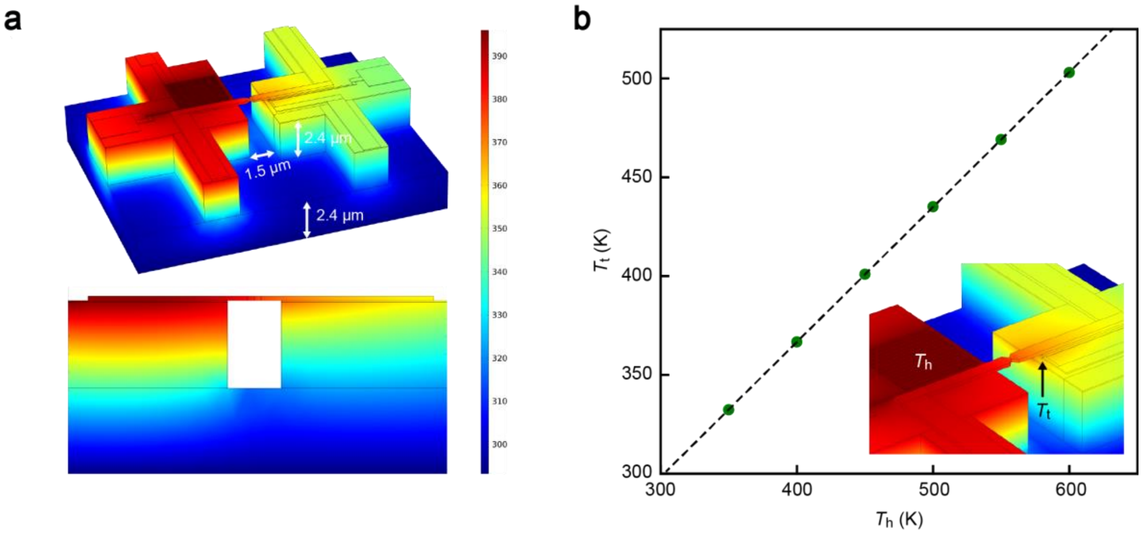

Figure S3. Numerical simulations of heat transport for thermocouple calibrations. a, Three dimensional model used for heat transport simulations using COMSOL package. The Au junction had 100 nm x 100 nm cross-section at the narrowest constriction mimicking the structure before the mechanical breakdown. The dimensions were extracted from SEM images. Colour maps show the temperature profile. Upper and bottom images are the inclined and the side views, respectively. **b,** Dependence of the temperature at the point contact of the Au and Pt nanowires on the heater temperature T_h.

2. Supplementary figures:

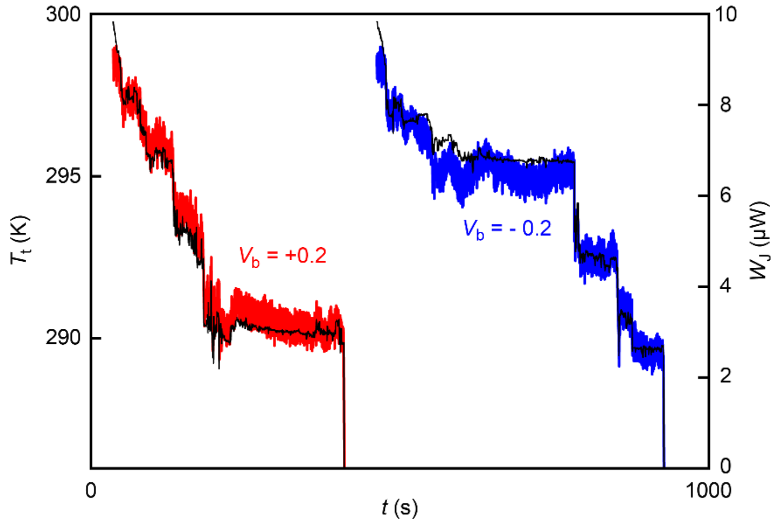

Figure S4. Power dependence of the thermocouple temperature. Direct comparison of the thermocouple temperature T_t (red: V_b = + 0.2 V; blue: V_b = - 0.2 V) with the input power W_J (black line) during junction breakdown. T_t is tracing the change in W_J, manifesting the predominant role of the remote heat dissipation on the thermocouple temperature.

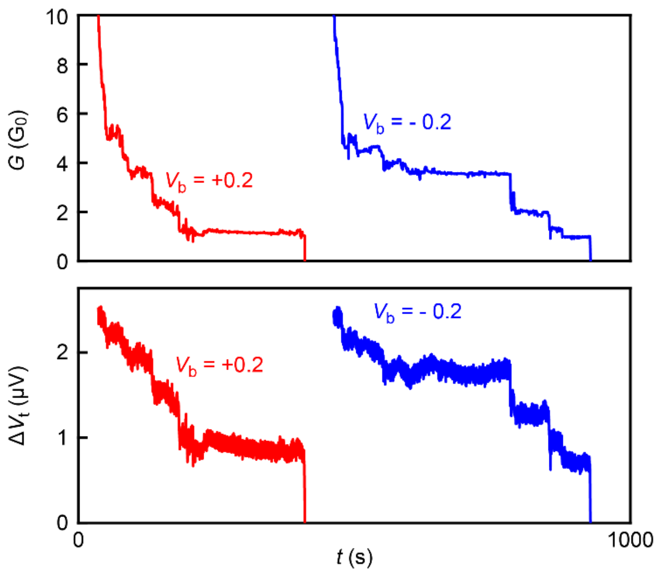

Figure S5. Conductance and thermocouple voltage traces at positive and negative bias voltage conditions. Conductance (G) drops in a stepwise manner upon junction elongation irrespective of the polarity of the applied voltage

V_b on the Au junctions (top). Meanwhile, the thermovoltage ΔV_t at the thermocouple changed synchronously to the G-steps (bottom).

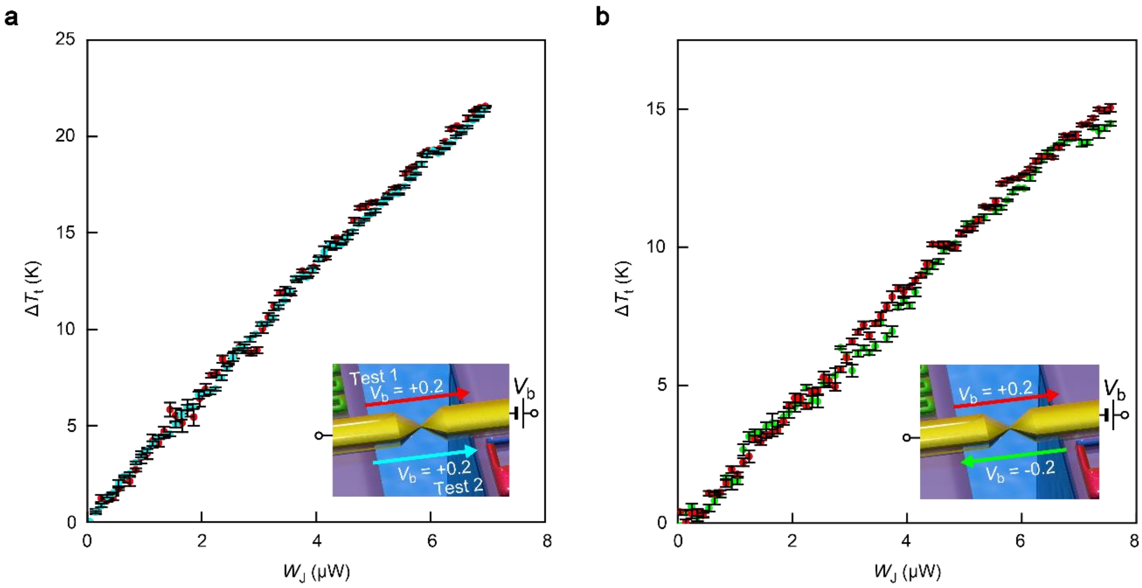

Figure S6. Voltage polarity dependence of the thermocouple temperature ΔT_c. a-b, ΔT_c plotted as a function of the input power W_J for the case of two independent measurements conducted under the same (a: V_b = + 0.2 V) or different bias conditions (b: V_b = ± 0.2 V).

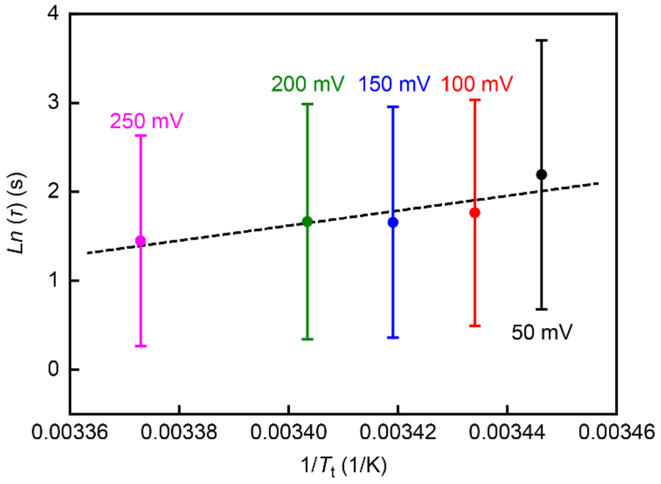

Figure S7. Semi-logarithmic plots of the average τ against the inverse thermocouple temperature T_t^{-1}. Dotted line is a linear fit that demonstrate non-notable influence of remote heating on the single-atom contact stability.

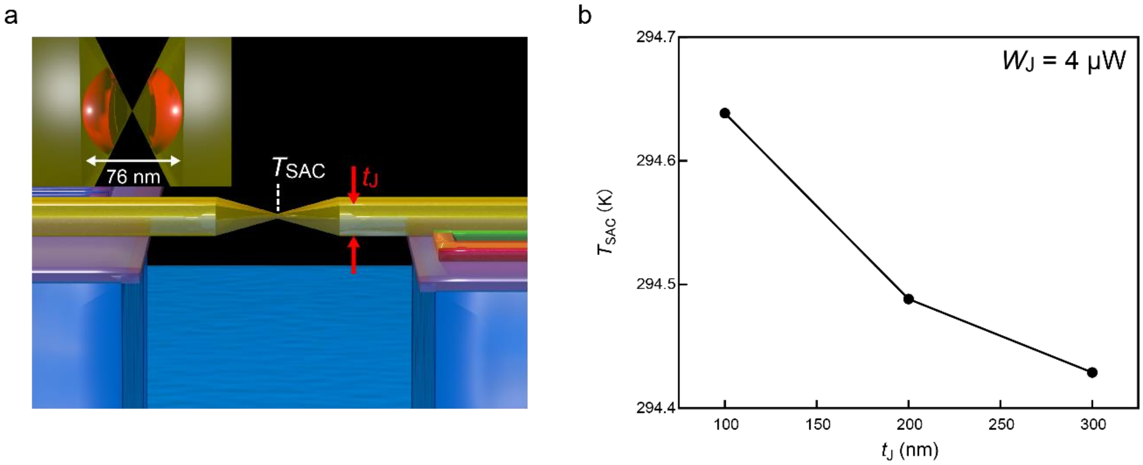

Figure S8. Effects of heat spreader on contact temperature. a, Schematic model used for the COMSOL simulation. The geometry of the thermocouple-embeded MCBJ was deduced from SEM images except the thickness t_J of the junction including the microleads, which was changed from 100 nm to 300 nm for studying the influence of contact cooling by thermal conduction. In the numerical analysis, the temperature T_{SAC} at the Au single-atom contact was estimated by assuming two heat sources at 38 nm away from there that mimics the remote heating taking place at the diffusive regions defined by the inelastic mean free path.[S2]

3. Supplementary reference

S1. Tsutsui, M., Morikawa, T., He, Y., Arima, A. & Taniguchi, M. High thermopower of mechanically stretched single-molecule junctions. *Sci. Rep.* 5, 11519 (2015).

S2. Heinrich, B., Tserkovnyak, Y., Woltersdorf, G., Brataas, A., Urban, R. & Bauer, G. E. W. Dynamic exchange coupling in magnetic bilayers. *Phys. Rev. Lett.* 90, 187601 (2003).
